# Optimized MLPA workflow for spinal muscular atrophy diagnosis: identification of a novel variant, NC_000005.10:g.(70919941_70927324)del in isolated exon 1 of *SMN1* gene through long-range PCR

**DOI:** 10.1186/s12883-024-03592-5

**Published:** 2024-03-11

**Authors:** Mei Yao, Liya Jiang, Yicheng Yu, Yiqin Cui, Yuwei Chen, Dongming Zhou, Feng Gao, Shanshan Mao

**Affiliations:** 1 Department of Neurology, Children’s Hospital, Zhejiang University School of Medicine, National Clinical Research Center for Child Health, Hangzhou, 310052 China; 2 Department of Infectious Diseases, Children’s Hospital, Zhejiang University School of Medicine, National Clinical Research Center for Child Health, Hangzhou, China; 3 Xiamen Biofast Biotechnology Co., Ltd., Xiamen, China; 4 Children’s Hospital, Zhejiang University School of Medicine, National Clinical Research Center for Child Health, Hangzhou, China

**Keywords:** Spinal muscular atrophy, Exon 1, Isolated, g.70919941_70927324del, Diagnosis

## Abstract

**Background:**

Spinal muscular atrophy (SMA) is a rare autosomal recessive hereditary neuromuscular disease caused by *survival motor neuron 1* (*SMN1*) gene deletion or mutation. Homozygous deletions of exon 7 in *SMN1* result in 95% of SMA cases, while the remaining 5% are caused by other pathogenic variants of *SMN1*.

**Methods:**

We analyzed two SMA-suspected cases that were collected, with no *SMN1* gene deletion and point mutation in whole-exome sequencing. Exon 1 deletion of the *SMN* gene was detected using Multiplex ligation-dependent probe amplification (MLPA) P021. We used long-range polymerase chain reaction (PCR) to isolate *the SMN1* template, optimized-MLPA P021 for copy number variation (CNV) analysis within *SMN1* only, and validated the findings via third-generation sequencing.

**Results:**

Two unrelated families shared a genotype with one copy of exon 7 and a novel variant, g.70919941_70927324del, in isolated exon 1 of the *SMN1* gene. Case F1-II.1 demonstrated no exon 1 but retained other exons, whereas F2-II.1 had an exon 1 deletion in a single *SMN1* gene. The read coverage in the third-generation sequencing results of both F1-II.1 and F2-II.1 revealed a deletion of approximately 7.3 kb in the 5’ region of *SMN1*. The first nucleotide in the sequence data aligned to the 7385 bp of NG_008691.1.

**Conclusion:**

Remarkably, two proband families demonstrated identical *SMN1* exon 1 breakpoint sites, hinting at a potential novel mutation hotspot in Chinese SMA, expanding the variation spectrum of the *SMN1* gene and corroborating the specificity of isolated exon 1 deletion in SMA pathogenesis. The optimized-MLPA P021 determined a novel variant (g.70919941_70927324del) in isolated exon 1 of the *SMN1* gene based on long-range PCR, enabling efficient and affordable detection of SMN gene variations in patients with SMA, providing new insight into SMA diagnosis to *SMN1* deficiency and an optimized workflow for single exon CNV testing of the *SMN* gene.

**Supplementary Information:**

The online version contains supplementary material available at 10.1186/s12883-024-03592-5.

## Background

Spinal muscular atrophy (SMA) is a neuromuscular genetic disorder associated with multiple system dysfunctions, including respiratory, digestive, and skeletal disorders, causing a substantially diminished quality of life for affected individuals [1–3]. The clinical classification, including types 0, I, II, III, and IV, is based on the age of onset and the maximum motor capacity that can be achieved. Types I, II, and III are prevalent in childhood [4]. Among them, type Ia SMA represented the most severe form, diagnosed by onset within three months of birth. Without medication and multidisciplinary management support, children with type Ia SMA typically obtained a survival time of < 2 years [5, 6].

SMA is a progressive neurodegenerative disease inherited as an autosomal recessive trait [7]. It affects 1 in 6000–10,000 newborns, with a carrier frequency of 1:40–80 in the general population [7, 8]. SMA is frequently caused by homozygous deletions of the *survival motor neuron 1* (*SMN1*) gene or by the deletion of one allele and the inheritance of a single mutant allele located on chromosome 5q11-q13.1 [9, 10]. *SMN2*, a paralog of *SMN1*, shares high sequence similarity except for a single-nucleotide polymorphism at NM_000344.3:c.840C > T in exon 7, which changes splicing and reduces the amount of full-length protein [9, 10]. Approximately 95% of SMA cases are caused by homozygous deletions of exon 7 in *the SMN1* gene, and the remaining is usually associated with compound heterozygous mutations that are challenging to identify using routine genetic testing methods [11].

In recent years, significant progress has been made in pharmacotherapeutics for SMA. Nusinersen, Risdiplam, and Zolgensma are the three disease-modifying treatments currently used in clinical practice [12, 13]. Extensive research has revealed that disease-modifying treatments effectively improve motor function and overall survival in children with SMA, and the early treatment initiation enables them to achieve motor milestones comparable to those of unaffected children [14–20]. Consequently, early disease diagnosis is particularly important in this context, especially considering the availability of therapeutic interventions.

The current diagnostic guidelines for SMA recommend that Multiplex ligation-dependent probe amplification (MLPA) testing for the *SMN* gene should be performed first in patients with high clinical suspicion of SMA [21, 22]. Long-range polymerase chain reaction (LR-PCR) combined with nested PCR and sequencing should be further conducted if only one copy of the *SMN1* gene is detected to determine if the patient carries the mutation and confirm if they are complex heterozygous patients. Despite such specific diagnostic guidelines, cases with highly suspected clinical phenotypes of SMA remain, but routine genetic testing fails to identify any variants in *SMN1*.

This study identified two cases of patients with SMA with homozygous deletion of isolated exon 1 while retaining one copy of *SMN1* exon 7, using optimized-MLPA P021 and LR-PCR, expanding the mutational profile of the *SMN1* gene. Both patients received prompt treatment after diagnosis, which improved or stabilized their motor function. Notably, this study emphasizes a novel pathogenesis variant in SMA, contributes valuable insights to the expanding landscape of SMA genetics, and reinforces the need for early diagnosis and intervention in the clinical management of SMA.

## Methods

### Case presentation

#### Family 1

Case F1-II.1 was the first child born to healthy, nonconsanguineous parents without a specific birth history. The child had an unsteady head at 3 months. He was hospitalized for severe pneumonia at 4 months and could not be taken off a ventilator during treatment. MLPA P021 SMA kit was used to detect the copy number of the *SMN* gene, and sequencing was used for this family. The copy number of *SMN1*:*SMN*2 in case F1-II.1 was 1:2, with his mother 2:1 and father 1:2. Meanwhile, whole-exome sequencing (WES) revealed no mutation. The child could not access timely multidisciplinary treatment because the SMA diagnosis could not be confirmed. The child experienced severe failure to thrive, recurrent aspiration pneumonia, and global developmental delay in motor function, as the disease progressed, eventually requiring tracheotomy ventilator support and a nasal feeding tube at a later stage.

#### Family 2

Case F2-II.1, a 2-month-old Chinese boy, was highly suspected of having SMA after contracting pneumonia but failed to wean from mechanical ventilation. Neurological examination and electromyography revealed severe symmetric muscle weakness and low muscular tension, indicating neurogenic damage. Liver and kidney function, serum electrolytes, or creatine kinase levels demonstrated no abnormalities. The result of MLPA P021 indicated that exons 7 and 8 in the *SMN1* gene had one copy, whereas exons 7 and 8 in the *SMN2* gene had two copies. *SMN1*:*SMN*2 genotype was 2:2 in his mother and 1:2 in his father. Moreover, whole-exome sequencing demonstrated no mutation. The boy was also delayed in the multidisciplinary team (MDT) because of undiagnosed SMA, and he was given nutritional support through a nasal feeding tube and put on mechanical ventilation.

### Genetic analysis

#### DNA sample extraction

Genomic DNA was extracted from peripheral lymphocytes in a 2-mL venous blood sample obtained from the patient and his family members. We added EDTA-K2 as an anticoagulant and extracted whole blood using a QIAamp DNA Mini Kit following the manufacturer’s instructions (Qiagen, Valencia, CA, USA).

#### Optimized-MLPA P021

*The SMN1* gene was specifically isolated from *SMN2* via long-range PCR from an exon 8-specific site, and the full length of the PCR product was purified through gel extraction (Qiagen, Valencia, CA, USA). Exon copy numbers of the *SMN1* gene were analyzed using the *SMN1* LR-PCR product by the MLPA P021 method, commercial kit from MRC-Holland. Additionally, the same approach was used to determine exon copy numbers of the *SMN2* gene. This approach incorporated Q-fragment, D-fragment, and other reference probes to ensure that no gDNA contamination interrupted the result, confirming that the normalized result exclusively represented copy number variants of *SMN1*. The genotype of *SMN1*:*SMN2* of 1:2 as the proband’s genotype was used as a standardized control in optimized-MLPA P021 to calculate each exon copy number. The size calling and allele recognition of the CE result were conducted with Coffalyser, and the copy numbers were normalized using Statistical Package for the Social Sciences.

#### SMN1 full-length long-range PCR

Amplification of full-length *SMN1* used a forward primer hybridized to the exon 1 transcription initiation site, along with a reverse primer specific to *SMN1*-specific exon 8. Specifically, we used the forward primer 5’-ATAGCTGAGCGTGGTGGCGCACGC-3’ and the reverse primer 5’-TGCTGGCCTCCCACCCCCACTC-3.’ PCR was performed using KOD FX Neo polymerase (Toyobo, Osaka, Japan) with 1 U of polymerase and 100 ng of genomic DNA to amplify a 28.2-kb region encompassing exons 1–8. LR-PCR was conducted according to the established protocol, with initial denaturation at 94℃ for 2 min, followed by five cycles of denaturation at 98℃ for 10 s; annealing and extension at 71.2℃ for 15 min, followed by five cycles of denaturation at 98℃ for 10 s; annealing and extension at 69.2℃ for 15 min, followed by five cycles of denaturation at 98℃ for 10 s; annealing and extension at 67.2℃ for 15 min, and 20 cycles of denaturation at 98℃ for 10 s; annealing and extension at 65.2℃ for 15 min, and a final extension at 65.2℃ for 7 min. The PCR product was verified using 0.7% agarose gel electrophoresis and extracted using a QIAEX II Gel Extraction Kit (Qiagen, Valencia, CA, USA). All gel extractions were performed following the instructions received from the manufacturers’ manuals.

#### Third-generation sequencing

Third-generation sequencing (TGS) was performed as previously described [23], using the same paired primers as those used in *SMN1* LR-PCR. This technique uses a closed, circular ssDNA template consisting of a double-stranded DNA insert with a single-stranded hairpin adapter at either end. DNA insertions can range in length from 1 kb to > 100 kb, allowing long sequencing read generation. A single polymerase is immobilized at the (transparent) bottom in each zero-mode waveguide, replicating a target DNA molecule. The incorporation of fluorescently labeled nucleotides generates a fluorescence signal upon excitation by a laser during the replication process, and a camera records the emission. The fluorophore is then cleaved from the nucleotide before incorporating the next dNTP. This process is repeated thousands of times to determine the identity and sequence of each basis in the SMRTbell template.

## Results

### Genetic study

#### Copy number of exons 1–8 of the SMN gene in MLPA P021

**Fig. 1:**
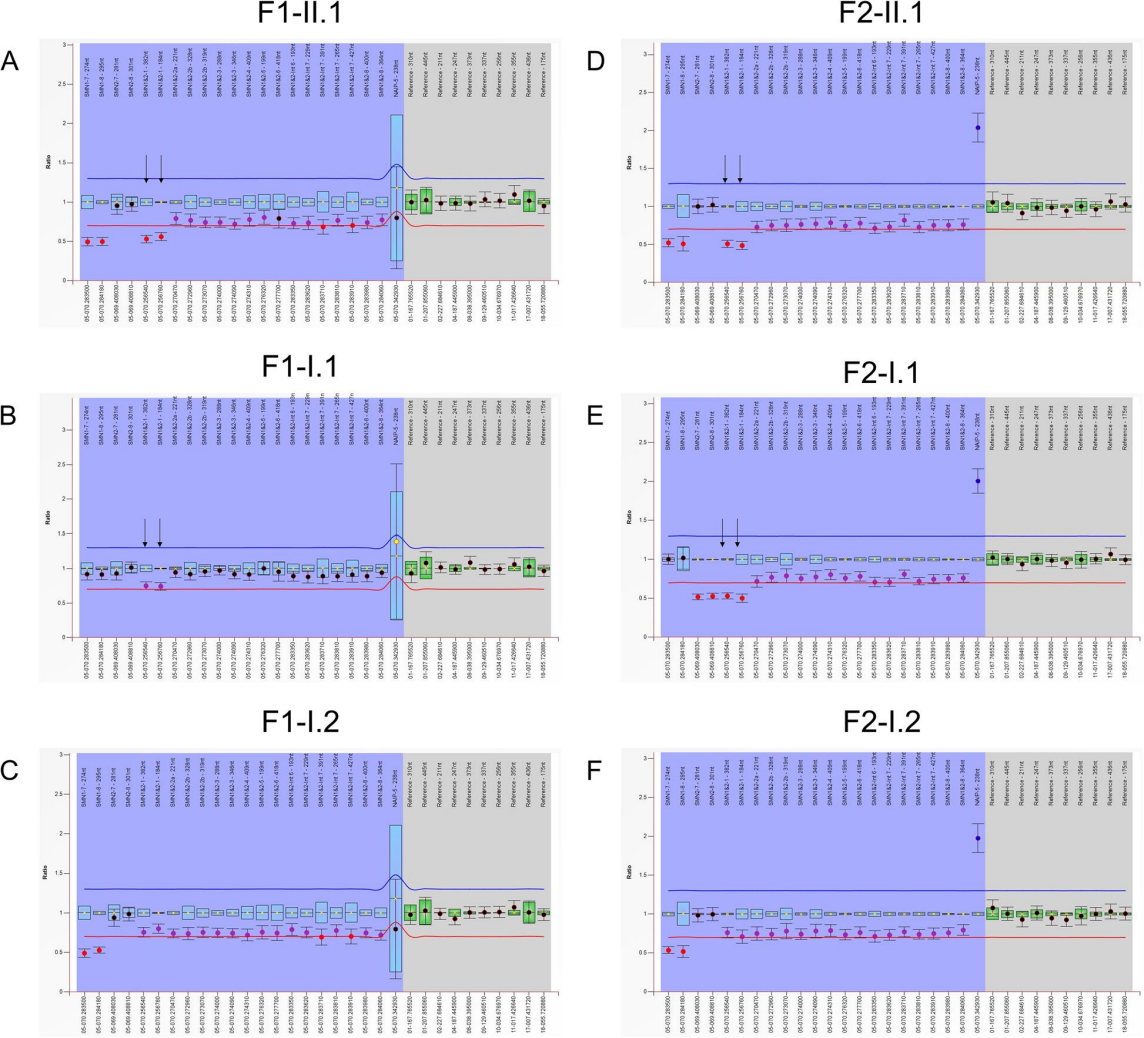
Results of MLPA P021 for both families. **a** Case F1-II.1 demonstrated two copy numbers of exon 1 and three copies of exons 2–6, while the exons 7 and 8 in survival motor neuron 1 (*SMN1*):*SMN2* copy number is 1:2. **b** The mother of F1-II.1 carries 2:2 copies of exons 7 and 8 in *SMN1* and *SMN2*; conversely, it carries two copies of exon 1 and four copies of exons 2–6. **c** Case F1-I.2, the father of proband, exhibited three copies of exon 1 and exon 2–6, and the *SMN1*:*SMN*2 copy number is 1:2. **d** Case F2-II.1 caused two copy numbers of exon 1 and three copies of exon 2–6, whereas the *SMN1*:*SMN*2 copy number is 1:2. **e** The mother of case F2-I.1, whose *SMN1*:*SMN2* copy number is 2:1, carries two copies of exon 1 and three copies of exons 2–6. **f** Case F2-I.2, the father of proband, exhibited three copies of exon 1 and exons 2–6, and the *SMN1*:*SMN2* copy number is 1:2

Case F1-II.1, in family 1, had two copies of exon 1 and three copies of exons 2–6. Further, the MLPA P021 of case F1-II.1’s mother, F1-I.1, had two copies of exon 1 and three copies of exons 2–6. The result indicated that case F1-II.1 and his mother have a single-copy deletion of exon 1, and F1-II.1’s father, F1-I.2, obtained three copies of the *SMN* gene without a single exon deletion. Case F2-II.1, in family 2, carries two copies of exon 1 and three copies of exons 2–6. His mother, F2-I.1, carries three copies of exon 1 and four copies of exons 2–6. Additionally, previous reports of WES and Sanger sequencing revealed no variants. Hence, we speculated that case F2-II.1 and his mother had a deletion of exon 1, and the father, F2-I.2, did not exhibit any single exon deletion (Fig. 1).

#### Optimized-MLPA P021 of exon 1 copy number in the SMN gene based on LR-PCR

Considering the identical exons 1–6, the copy numbers of exons 1–6 were detected using the conventional MLPA P021 as the sum of the *SMN* gene rather than the respective profiles of *SMN1* and *SMN2*. This study used LR-PCR to amplify *SMN1* and *SMN2* genes and used optimized-MLPA P021 the copy numbers of *SMN1* and *SMN2* genes were quantified using. The full length of *SMN1* was amplified from the upstream region of exon 1 to exon 8 c.1155G/A, and electrophoresis confirmed full-length amplification. The *SMN1* gene optimized-MLPA P021 result detected no *SMN2*-specific signals. The *SMN2*-specific signal length is 281 bp, indicating that only the *SMN1* gene was independently amplified. The copy numbers of exons in the optimized MLPA represent the real copy numbers of SMN1 without any interference from *SMN2*.

**Fig. 2:**
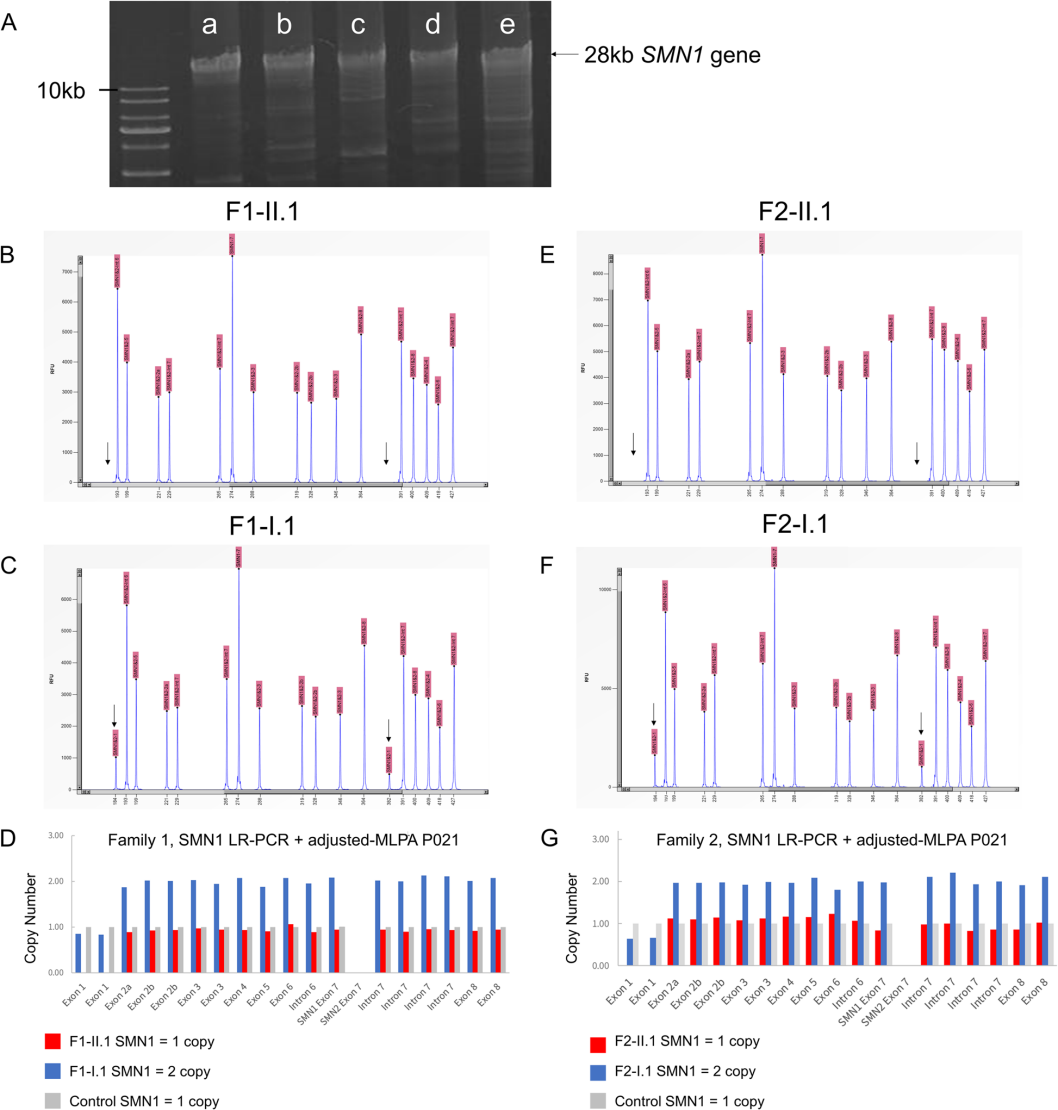
*SMN1* gene long-range PCR and optimized-MLPA P021 results for both probands and their mothers. **a** The full length of *SMN1*-specific amplification includes exons 1–8 c.1155 examined by electrophoresis. **b** The MLPA result of proband F1-II.1 indicated that none of the two probes of *SMN1* exon 1 (184 bp and 382 bp) was detected by PCR-CE, marked by arrows. Other probes from exons 2–7 were labeled on top. **c** The MLPA result of F1-I.1, mother of the proband, the two probes of *SMN1* exon 1 were marked by arrows. **d** Family 1’s copy numbers of each exon in the *SMN1* gene. **e** The MLPA result of F2-II.1, none of the *SMN1* exon 1 probe was detected, marked by arrows. **f** The MLPA result of the mother, F2-I.1, two of *SMN1* exon 1 signals were marked by arrows

Based on the optimized-MLPA P021 results in family 1, case F1-II.1 demonstrated zero copies of exon 1 and one copy of other exons in the *SMN1* gene, whereas his mother carried one copy of exon 1 within two copies of *SMN1*. Moreover, he carried two copies of exon 1 in the *SMN2* gene, whereas his mother had one copy. Case F2-II.1 had a deletion of exon 1 in the single *SMN1* gene, whereas his mother expressed one copy of exon 1 in the two copies *of the SMN1* gene (Fig. 2, supplementary Fig. 1). Both he and his mother carried two copies of exon 1 in the *SMN2* gene. The same procedure confirmed the *SMN2* exon copy number and no exon 1 deletion in the *SMN2* gene (data not shown).

The results of the study confirmed the absence of exon 1 in *SMN1* in both cases. Additionally, both cases inherited an allele with missing exon 1 from their respective mothers. They demonstrated a heterozygous deletion of exon 1 in the *SMN1* gene.

#### Third-generation sequencing (TGS) results of two cases

**Fig. 3:**
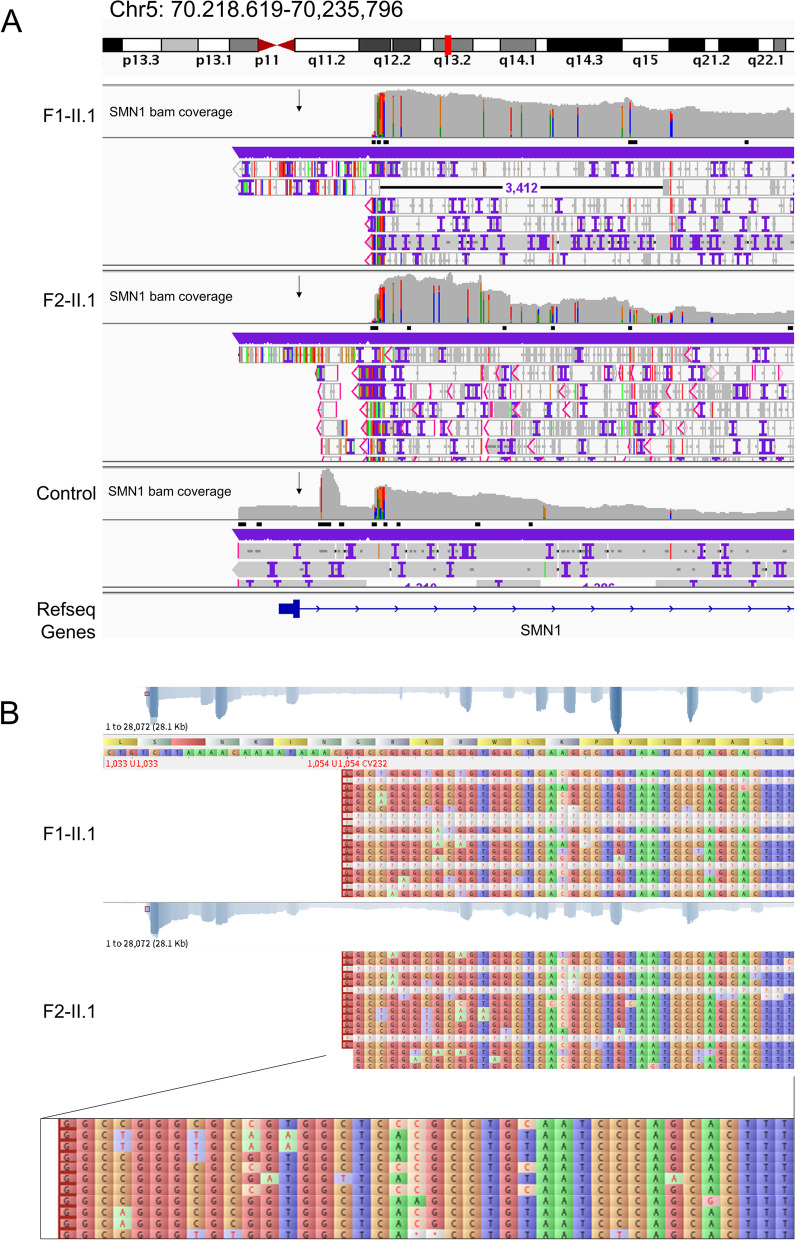
Third-generation sequencing (TGS) result of cases F1-II.1 and F2-II.1. **a** The IGV of TGS result of cases F1-II.1, F2-II.1, and the control obtained one copy of *SMN1*. A 7.3 kb region without any *SMN1* reads mapped on it was observed after the *SMN1* reads coverage in cases F1-II.1 and F2-II.1, and the region in the control group had read distributions. **b** Cases F1-II.1 and F2-II.1’s mapping sequences result generated by Tablet, and the sequences near the novel *SMN1* exon 1 deletion hotspot, NG_008691.1:g.7385

The TGS results of the SMN1 LR-PCR product were mapped to reference NG_008691.1. The read coverage in the TGS results of both F1-II.1 and F2-II.1 revealed a deletion of approximately 7.3 kb in the 5’ region of SMN1. The sequence data revealed that the first nucleotide aligned to the 7385 bp of NG_008691.1. Therefore, this SMN1 exon 1 deletion variant is designated as NC_000005.10: g.70919941_70927324del, a large deletion and novel pathogenic variant associated with SMA. The sequence of breakpoint junction was aligned with the Alu-repetitive elements using BLAST and revealed a high identity of 85% (Supplementary Fig. 2). SMN1 exon 1 is positioned at NG_008691.1:g.5001_5244, spanning a total length of 244 bp, and exon 2a is at g.18899_18970. Consequently, this newly determined large deletion encompasses the entire exon 1, and both cases have a defective SMN1 gene without exon 1 but retain a complete exon 2 (Fig. 3).

### Treatment and follow-up

Both patients underwent MDT and disease-modifying drug treatment after their diagnosis. Nusinursen was intravenously administered in case F1-II.1 and oral ribodiplam in case F2-II.1. Both patients demonstrated that their motor function remained stable or improved after treatment compared with their pretreatment condition. Respiratory function improved after treatment and blood oxygen saturation remained stable within 1 h of ventilator disconnection. Additionally, the patients demonstrated increased mobility, including rollover in motion, and enhanced limb movements.

## Discussion

This study described two cases of SMA with the g.70919941_70927324del variant of isolated exon 1 of the *SMN1* gene, which has not been previously reported. The patients from two unrelated families demonstrated clinical symptoms that strongly indicated SMA, and MLPA P021 revealed a single-copy number of *SMN1*. Whole exon sequencing yielded negative results, which delayed diagnosis and treatment. In these cases, the combination of LR-PCR and optimized-MLPA P021 along with TGS techniques was used to investigate and elucidate the underlying pathogenic mechanisms. Our results contribute to the expansion of the variation profile of SMA pathogenic genes to enhance SMA diagnosis accuracy and treatment, ultimately improving patients’ survival rate by increasing the detection rate of complex heterozygous mutations.

SMA is a rare, progressive neurodegenerative disease caused by homozygous deletion or mutation of the *SMN1* gene on chromosome 5q13 [2]. SMA stands as the leading cause of infant genetic mortality with the prevalence of SMA being approximately 1 in 10,000 live births [3–5]. The homozygous deletion of the *SMN1* gene is responsible for 95% of patients with SMA, and the remaining 5% demonstrated compound heterozygous [3–5]. The diagnostic guidelines clearly state that MLPA testing should be performed first in patients with clinically suspected SMA. Further investigation using LR-PCR combined with Sanger sequencing should be performed to identify the presence of potential mutations if the detection results indicate the presence of heterozygous *SMN1* [21, 22]. Undiagnosed cases, except for homozygous deletions and complex heterozygosity of SMA, remain with highly suspected SMA by routine genetic testing, which frequently causes misdiagnosis, missed diagnosis, and delayed treatment.

Two cases initially underwent genetic testing based on diagnostic guidelines for SMA. The MLPA P021 analysis indicated that both cases had one copy of exons 7 and 8 in the *SMN1* gene and two copies of exons 7 and 8 in the *SMN2* gene. Meanwhile, the WES revealed no mutation in either case. MLPA P021 analysis indicated that a couple of mothers shared the same genotypes, with the copy number of exon 1 being less than that of exons 2–6. These interesting results initially revealed the presence of an exon 1 deletion in the *SMN* gene in patients and their mothers. In this disease, SMA is exclusively caused by variants in the *SMN1* gene. Hence, we still need to isolate the *SMN1* gene for a separate detection to make a definitive diagnosis, as MLPA P021 alone could not determine the presence of axon 1 in the *SMN1* gene.

LR-PCR is an efficient and broadly applicable method that is frequently used to identify intragenic *SMN1* or *SMN2* mutations [24, 25]. Kubo et al. used this method to detect intragenic mutations in *SMN1* and hybrid *SMN* genes [24]. LR-PCR was used to amplify the *SMN1* and *SMN2* genes of case F1-II.1, case F2-II.1, and their mothers, respectively, to confirm that the deletion of exon 1 is in the *SMN1* gene. We then used optimized-MLPA P021 to quantify each exon copy number of the *SMN1* and *SMN2* genes. Cases F1-II.1 and F2-II.1 demonstrated homozygous deletion of exon 1 in SMN1, which was 0 + 1^△exon1^. Their mothers carried one copy number of exon 1 of the *SMN1* gene, all of which were 1 + 1^△exon1^. Hence, the *SMN1* gene in patients with exon 1 deletion is inherited from the mother, resulting in SMA. Furthermore, both cases with a single homozygous deletion of exon 1 had one copy number of exon 7 in the *SMN1* gene based on the LR-PCR and optimized-MLPA P021 technique.

This study used the TGS technologies, which are based on single-molecule sequencing and real-time sequencing, to ensure the accuracy and reliability of the results [23, 26]. A recent study revealed that TGS identified the copy numbers of *SMN1* and *SMN2*, intragenic mutations, and potential silent carriers [27]. Two patients underwent TGS to ensure the rigor and accuracy of the results. The TGS revealed that the length of the missing breakpoints in both cases spanned a length of 7384 bp of *SMN1*, both of which are larger than the length of exon 1. Both cases are with SMA with the g.70919941_70927324del variant of isolated exon 1 in the *SMN1* gene, which has not been previously reported. Previous studies have revealed that some patients with SMA had one deletion of the entire *SMN1* and one deletion of exons 1–6 in *SMN1* [28], and some carried the heterozygous deletion of *SMN1* with a large Alu-mediated deletion involving exons 5 and 6 [29]. Zhang et al. determined two types of partial deletions of *SMN1* in seven patients, one is an isolated deletion of exon, while the other ones were caused by codeletions of exons 1, 4, and 7 [30]. Recent advancements in Ultra-LRS technology have determined two patients with SMA with different breakpoints in detecting exon 1 of the *SMN1* gene [31]. Notably, these two cases demonstrated distinct genotypes, indicating the genetic heterogeneity that can exist within exon 1 deletion. Ultra-LRS has been proven as a valuable tool in identifying such variations, and considering the limitations of this approach is important, particularly the high costs, complex procedures, and time-consuming nature of its implementation. Routine diagnostic workflows frequently require a more accessible and cost-effective solution that can be widely used to accurately diagnose SMA. Considering these previous results, the deletion of other exons, such as exon 7, alongside the absence of exon 1, is crucial. The new finding of the g.70919941_70927324del variant, an isolated exon 1 deletion in the *SMN1* gene, may represent a hotspot mutation in Chinese patients with SMA.

A wide range of phenotypic manifestations are observed in SMA, encompassing severely affected neonates (type 0) to adults displaying minimal symptoms (type IV). This variation can be ascribed to the number of SMN2 copies and the quantity of full-length SMN protein generated by each patient, which is further influenced by potential disease modifiers that affect the ultimate phenotype. Our study revealed that two patients with SMA demonstrated a genotype characterized by the presence of one copy of *SMN1* and two copies of *SMN2*. These individuals exhibited a homozygous deletion of exon 1 in the SMN1 gene, specifically identified as 0 + 1^△exon 1^. Two patients presented with an onset age of 3 months and were both diagnosed with SMA type Ia. Typically, SMA type Ia is associated with a severe clinical phenotype and significantly reduced life expectancy. However, the survival time of these particular two patients was extended for over a year. Previous research has revealed that intragenic SMN1 mutations cause clinical phenotypes of varying severity, regardless of the number of SMN2 copies [32]. We speculate that patient who possess compound heterozygous mutations with the presence of exon 1 deletion may exhibit relatively milder symptoms compared to those with homozygous deletions in *SMN1* gene. Further research is required to validate this hypothesis by examining more samples.

The diagnosis procedure in our study is described below. MLPA021 was initially used to determine the copy number of each exon, and subsequently, LR-PCR and optimized MLPA021 were used to test the single number of exons in *SMN1* and *SMN2*. TGS was performed to prove the detailed breakpoint. LR-PCR was usually prescribed to determine *SMN1* point mutations but was never used to test the copy number variant of each exon previously [26]. The strength of the present study is that we combined LR-PCR with optimized MLPA021 to detect single exon deletion for the first time. This approach provided several advantages, including validation of *SMN1* isolation, detection of gDNA contamination, and copy number variant testing for each exon. TGS technologies accurately identify genetic variations, but they are associated with high costs and may not be easily accessible for widespread use. Therefore, the combined use of optimized MLPA021 and LR-PCR provides a practical and cost-effective alternative for verifying single exon deletions in clinical practice.

The g.70919941_70927324del variant of isolated exon 1 in *the SMN1* gene has never been previously reported. Previous research has determined two patients with SMA who demonstrated distinct breakpoints in exon 1 deletion within the SMN1 gene [31]. Utilizing BLAST to locate the breakpoint junction, we observed that the breakpoint ends precisely align with Alu-repetitive elements. Subsequent analysis unveiled consensus sequences of Alu elements, demonstrating a notable alignment, attaining a high identity of 85%. This robust evidence suggests the potential involvement of Alu-mediated deletions, specifically through Alu recombination-mediated deletion (ARMD). This discovery implies a potential hotspot for SMN1 exon 1 deletion linked to Alu-mediated rearrangements. Our study included cases F1-II.1 and F2-II.1, both of which possess the same genotype, thereby providing further confirmation that homozygous deletion of exon 1 may represent a potential hotspot for SMA. We plan to address whether this variant is a hotspot and conduct studies of large-scale samples to assess its carrying rate. The results of this study raised serious concerns about the other exons in the *SMN1* gene, not just exons 7 and 8. The current SMA diagnosis consensus and guidelines primarily focus on exon 7 deletion but do not include other exons, and the *SMN1* gene can only be distinguished from SMN2 when some mutation is suspected to be carried by the patient. However, our study raises concerns about the potential involvement of other exons in *SMN1*. The framework developed in this study can be used as a reference for patients who are clinically suspected of having SMA but cannot be diagnosed by routine testing. We developed an optimized workflow for SMA diagnosis, and the optimized-MLPA P021 combined with LR-PCR obtained a better economic benefit compared with the high cost and low penetration of the TGS method (Table 1). Furthermore, this optimized workflow can be widely implemented by any laboratory using PCR technology. Our improved diagnostic methods provide advantages in terms of speed, sensitivity, reliability, and feasibility. We can improve diagnosis accuracy under the current guidelines for SMA diagnosis, which achieves the goals of earlier treatment for elongating the life span of patients and improving their quality of life, by incorporating our framework into the current guidelines for SMA diagnosis. Overall, our study reveals a completely novel mutation pattern in the *SMN1* gene that causes SMA, providing valuable insights into the underlying causes of the disease. Additionally, our steady diagnostic workflow for *SMN1* mutations served as a reference for clinically suspected patients with SMA who cannot be diagnosed through routine testing.

**Table 1:** Characteristic and comparison of *SMN1* single exon CNV detection via TGS and optimized MLPA

**Parameters**	**TGS**	**Optimized-MLPA**
Fundamental technology	Single-molecule real-time sequencing	Relative quantification and fragment analysis
Analysis procedure	LR-PCR + TGS	LR-PCR + MLPA
Duration	2 days	2 days
Cost	$450–$850 per test [33]	$29 per test
Data analysis	Complex	Routine Use

## Conclusion

Our results emphasize the significance of this homozygous isolated deletion of exon 1 in the *SMN1* gene g.70919941_70927324del as a significant pathogenic variant for SMA. The isolated exon 1 copy number variation (CNV) is a previously unreported CNV, which expands the existing *SMN1* variants database and highlights the importance of considering the copy number variation of other exons within *SMN1*. Optimized-MLPA P021 may be an important complement to the current diagnostic workflow of SMA. We can greatly enhance the accuracy of diagnosis and reduce the likelihood of misdiagnosis by incorporating this optimized-MLPA assay. Early identification of cases with this specific exon 1 deletion allows for prompt treatment initiation, ultimately improving prognosis and survival rates for affected individuals.

### Supplementary Information

**Supplementary Material 1.**

**Supplementary Material 2.**

## Data Availability

The datasets generated during the current study are available in the NCBI repository [SRR26448352 and SRR26448353].

## Abbreviations

SMA: Spinal muscular atrophy
MLPA: Multiplex ligation-dependent probe amplification
MDT: Multidisciplinary team
TGS: Third-generation sequencing
WES: Whole-exome sequencing
LR-PCR: Long-Range PCR
Ultra-LRS: Ultra-long read sequencing
