# Supplementary material for: Optimized MLPA workflow for spinal muscular atrophy diagnosis: identification of a novel variant, NC_000005.10:g.(70919941_70927324)del in isolated exon 1 of SMN1 gene through long-range PCR
Source: BMC Neurol. 2024 Mar 11;24:93. doi: 10.1186/s12883-024-03592-5 (PMC10926642; doi:10.1186/s12883-024-03592-5)
Supplement: Supplementary file 1 — Supplementary Material 1. [file 12883_2024_3592_MOESM1_ESM.pdf]

Optimized MLPA Workflow for Spinal Muscular Atrophy Diagnosis: Identification of a Novel Variant, NC_000005.10:g.(70919941_70927324)del in Isolated Exon 1 of *SMN1* Gene through Long-Range PCR

Mei Yao[1,2], Liya Jiang[1], Yicheng Yu[1], Yiqin Cui[1], Yuwei Chen[3], Dongming Zhou[4], Feng Gao[1], Shanshan Mao [1*]

1. Department of Neurology, Children's Hospital, Zhejiang University School of Medicine, National Clinical Research Center for Child Health, Hangzhou, China.

2. Department of Infectious Diseases, Children's Hospital, Zhejiang University School of Medicine, National Clinical Research Center for Child Health, Hangzhou, China.

3. Xiamen Biofast Biotechnology Co., Ltd., Xiamen, China

4. Children's Hospital, Zhejiang University School of Medicine, National Clinical Research Center for Child Health, Hangzhou, China.

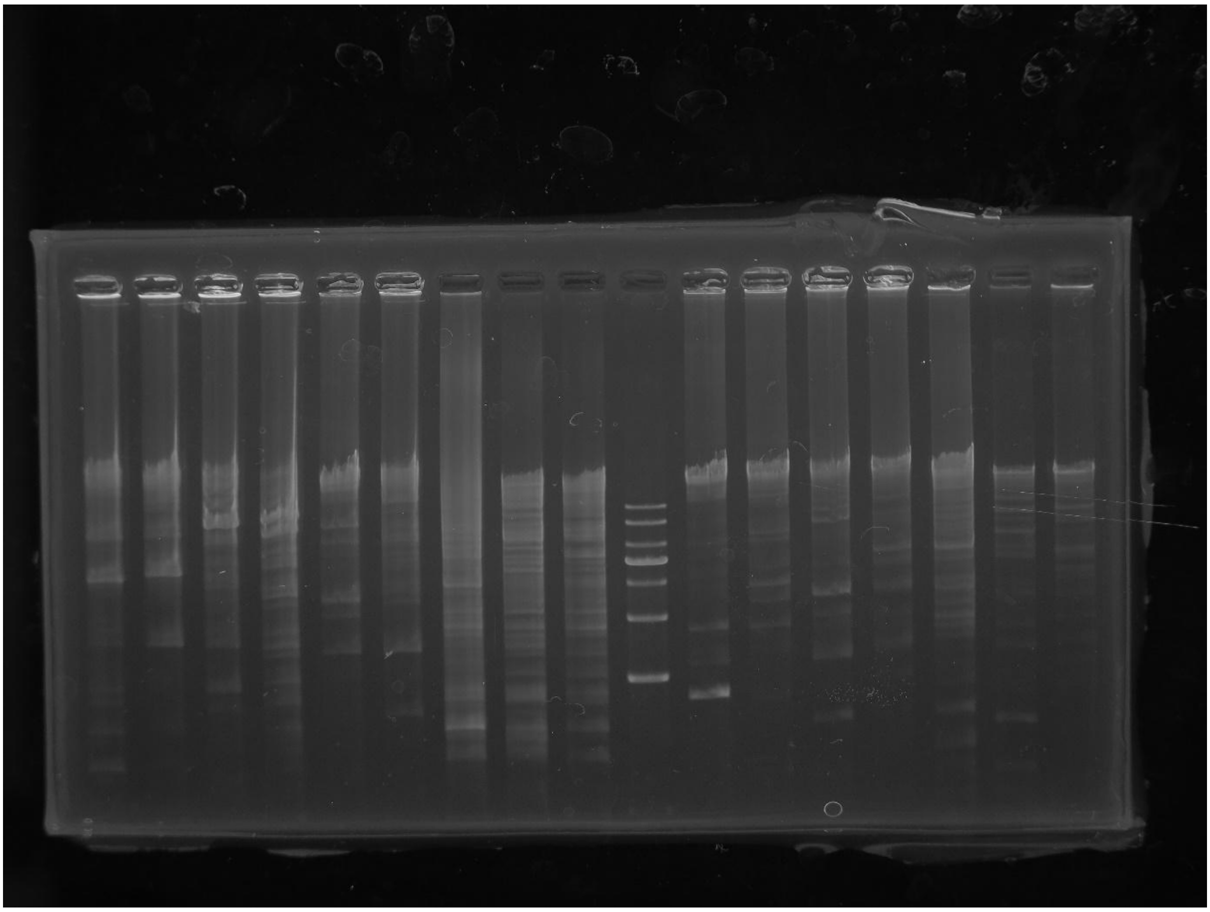

Supplementary Figure 1: Full-length gels of original Figure 2A
