# Supplementary material for: Optimized MLPA workflow for spinal muscular atrophy diagnosis: identification of a novel variant, NC_000005.10:g.(70919941_70927324)del in isolated exon 1 of SMN1 gene through long-range PCR
Source: BMC Neurol. 2024 Mar 11;24:93. doi: 10.1186/s12883-024-03592-5 (PMC10926642; doi:10.1186/s12883-024-03592-5)
Supplement: Supplementary file 2 — Supplementary Material 2. [file 12883_2024_3592_MOESM2_ESM.pdf]

A

```
Identities:249/293(85%),  Gaps:11/293(3%), Strand: Plus/Minus

Alu     12   GCCGGGCGCGGTGGCTCACGCCTGTAATCCCAGCACTTTGGGAGGCCGAGGCGGGCGGAT  71
             ||||||||| |||||||||||||||||||||||||||||  ||||| |||| | || || 
Sample  290  GCCGGGCGCAGTGGCTCACGCCTGTAATCCCAGCACTTTCAGAGGCTGAGGTGAGCAGAA  231

Alu     72   CAC--GAGGTCAGGAGATCGAGACCGTCCTGGCTAACACGGTGAAACCCC-----GTCTC  124
             |||  || |||||||| || |||||  |||||| |||| |||||||||||      ||||
Sample  230  CACTTGATGTCAGGAGTTCAAGACCAGCCTGGCCAACATGGTGAAACCCCACCCCATCTC  171

Alu     125  TACTAAAA-TACaaaaaaaTTAGCCGGGCGTGGTGGCGGGCGCCTGTAGTCCCAGCTACT  183
             |||||||| |||  |||||||||| ||||||||||||||||||||||| ||||||||| |
Sample  170  TACTAAAAATAC--AAAAATTAGCTGGGCGTGGTGGCGGGCGCCTGTAATCCCAGCTATT  113

Alu     184  CGGGAGGCTGAGGCAGGAGAATGGCGTGAACCCGGGAGGCGGAGCTTGCAGTGAGCCGAG  243
             | |||||||||||| |||||||  | ||| ||| |||||||||| |||||||||||||||
Sample  112  CAGGAGGCTGAGGCTGGAGAATCACTTGA-CCCTGGAGGCGGAGGTTGCAGTGAGCCGAG  54

Alu     244  ATCGCGCCACTGCACTCCAGCCTGGGCGACAGAGCGAGACTCCGTCTCaaaaa  296
             ||| | ||||| ||||||||| ||| | |||||| ||||||||||||||||||
Sample  53   ATCACACCACTACACTCCAGCTTGGACAACAGAGTGAGACTCCGTCTCAAAAA  1
```

B

```
Identities:251/296(85%),  Gaps:3/296(1%), Strand: Plus/Plus

Alu     11   GGCCGGGCGCGGTGGCTCACGCCTGTAATCCCAGCACTTTGGGAGGCCGAGGCGGGCGGA  70
             ||||||||||||||||||||||||||||||||||||||||||||| | |||| ||| |||
Sample  1    GGCCGGGCGCGGTGGCTCACGCCTGTAATCCCAGCACTTTGGGAGACTGAGGTGGGTGGA  60

Alu     71   TCAC--GAGGTCAGGAGATCGAGACCGTCCTGGCTAACACGGTGAAACCCCGTCTCTACT  128
             | ||  ||||||||||| | ||||||  ||||||  | | || |||||||| |||||| |
Sample  61   TTACCTGAGGTCAGGAGTTTGAGACCAACCTGGCCGATATGGCGAAACCCCATCTCTAAT  120

Alu     129  -AAAATACaaaaaaaTTAGCCGGGCGTGGTGGCGGGCGCCTGTAGTCCCAGCTACTCGGG  187
              |||||||| ||||||||||||||  ||||||| || ||||||| ||||||||||||  |
Sample  121  AAAAATACAGAAAAATTAGCCGGGAATGGTGGCAGGTGCCTGTAATCCCAGCTACTCAAG  180

Alu     188  AGGCTGAGGCAGGAGAATGGCGTGAACCCGGGAGGCGGAGCTTGCAGTGAGCCGAGATCG  247
             |||||| |||||||| ||  | || |||| ||||| |||| ||||||||||||||||||
Sample  181  AGGCTGTGGCAGGAGTATCCCTTGGACCCAGGAGGTGGAGGTTGCAGTGAGCCGAGATCA  240

Alu     248  CGCCACTGCACTCCAGCCTGGGCGACAGAGCGAGACTCCGTCTCaaaaaaaaaaaa  303
             |||||||| ||||||||||||| ||| | || |||||| |  |||||||||||||||
Sample  241  CGCCACTGTACTCCAGCCTGGACGATATAGTGAGACTTCACCTCAAAAAAAAAAAA  296
```

Supplementary Figure 2. Alignment of Breakpoint Junction with Alu-Repetitive Elements.

(A) BLAST analysis showed the alignment of the left breakpoint junction sequence with Alu-repetitive elements. (B) The left breakpoint junction sequence aligned with Alu-repetitive elements, highlighting a substantial identity of 85% in both alignments.
